# Evaluation of the Effect of Menisci on Tibial Slope and the Correlation With Body Mass Index

**DOI:** 10.7759/cureus.72633

**Published:** 2024-10-29

**Authors:** Iskender Yilmaz, Sevda Lafci Fahrioglu, Ozum Yuksel Bugdayci, Sezgin Ilgi

**Affiliations:** 1 Anatomy, University of Kyrenia, Kyrenia, CYP; 2 Anatomy, Cyprus International University, Nicosia, CYP; 3 Radiology, Cyprus International University, Nicosia, CYP; 4 Anatomy, Near East University, Nicosia, CYP

**Keywords:** knee anatomy, knee instability, knee mri, meniscal slope, posterior tibial slope

## Abstract

Background

One of the important factors affecting the biomechanics of the knee joint is the posterior tibial slope which is the tibial plateau’s anatomical inclination toward the posterior of the sagittal plane. This inclination, which affects anterior-posterior stability, is important for the kinematics of the knee joint. Changes in the tibial slope may cause a deficit in the stability and function of the knee joint. We aimed to examine the inclination of the posterior horn of the meniscus and posterior tibial slope in healthy individuals and investigate the effect of body mass index on these measurements.

Methodology

A total of 34 magnetic resonance images and lateral knee radiographs were evaluated in this study. The study included individuals aged 15 to 78 without a history of previous injury or surgery of their knee.

Results

In the measurements made on magnetic resonance images, a statistically significant difference was found between 25% lateral meniscus slope (mean ± SD = 28.08 ± 1.88) and 25% medial meniscus slope (mean ± SD = 27.31 ± 1.41) (p = 0.05). At the same time, a statistically significant difference (p = 0.011) was found between 25% medial combined slope (mean ± SD = 29.05 ± 3.80) and 25% lateral combined slope (mean ± SD = 30.62 ± 2.99). There was no statistically significant difference between tibial and meniscus slopes, body mass index, gender, and age.

Conclusions

Our study results have shown that the 25% lateral meniscus and combined slopes are greater than the 25% medial meniscus slope.

## Introduction

The knee joint, articulatio genus, is the largest synovial joint in the human body formed by the medial and lateral condyles of the femur and tibia, along with the posterior surface of the patella, connected by ligaments and the joint capsule [1]. This joint plays an important role in daily life activities, such as transferring body weight and appropriately distributing momentum in the walking pattern [2]. The stability of the joint during movement is maintained by ligaments, the joint capsule, muscles, and tendons [3]. Along with these structures, the posterior tibial slope (PTS) and menisci also contribute to joint stabilization [4,5].

PTS is defined as the angle between the line drawn parallel to the proximal joint surface of the tibia (os tibia) from front to back and the proximal anatomical axis of the tibia [6]. An increase in this angle enhances the anterior-posterior laxity of the knee joint, resulting in greater movement and making stabilization more difficult [7,8]. Previous studies have shown that injuries to the medial meniscus lead to increased anterior-posterior tibial translation, while the lateral meniscus serves as a secondary stabilizer. A decrease in the function of these structures negatively affects joint stabilization [9,10]. Impairment of these factors compromises knee joint stability, negatively influencing body movements. Therefore, considering these factors in daily activities and rehabilitation could improve treatment outcomes.

Radiography is a readily accessible and valuable tool for differential diagnosis in skeletal system pathologies [11]. As plain radiography is inadequate for soft tissue evaluation, magnetic resonance imaging (MRI) is utilized, which enables detailed, multidimensional examination of tissues such as the menisci [12]. In this study, we aimed to evaluate the PTS using lateral radiographs and both PTS and meniscal slopes through MRI. Additionally, we analyzed the correlation between our findings and variables such as age, gender, and body mass index (BMI).

This manuscript was presented orally at the European Association of Clinical Anatomy-International Symposium of Clinical and Applied Anatomy Joint Congress, Padova University, Italy from September 14-16, 2021. This manuscript was also presented orally at the 22nd National Anatomy Congress, Turkey (online). This manuscript is produced from the thesis labeled “Menıscus medıalıs ve menıscus lateralıs’ın cornu posterıor’u ve posterıor tibial eğim arasındaki ilişkinin incelenmesi: direkt grafi ve MRG çalışması in 2021, Nicosia.”

## Materials and methods

Lateral radiographs and sagittal MRI scans from 32 healthy patients (34 knees) without meniscal pathology, bone pathology, or previous surgery were evaluated in this study. The database was queried for all knee MRI scans conducted between January 2021 and April 2021 in a university hospital. Patients aged between 15 and 78 who had knee X-rays and MRIs reported as intact by the radiologist were included in the study. Patient records and radiology archive images were reviewed to ensure compliance with the defined criteria for MRI scans. Knees showing radiological evidence of meniscal degeneration, any ligament injury, or Grade II-IV osteoarthritis were excluded from the study. Ethical approval was obtained from the Near East University Scientific Research Ethics Board (approval number: 2021/90-1314).

**Figure 1:**
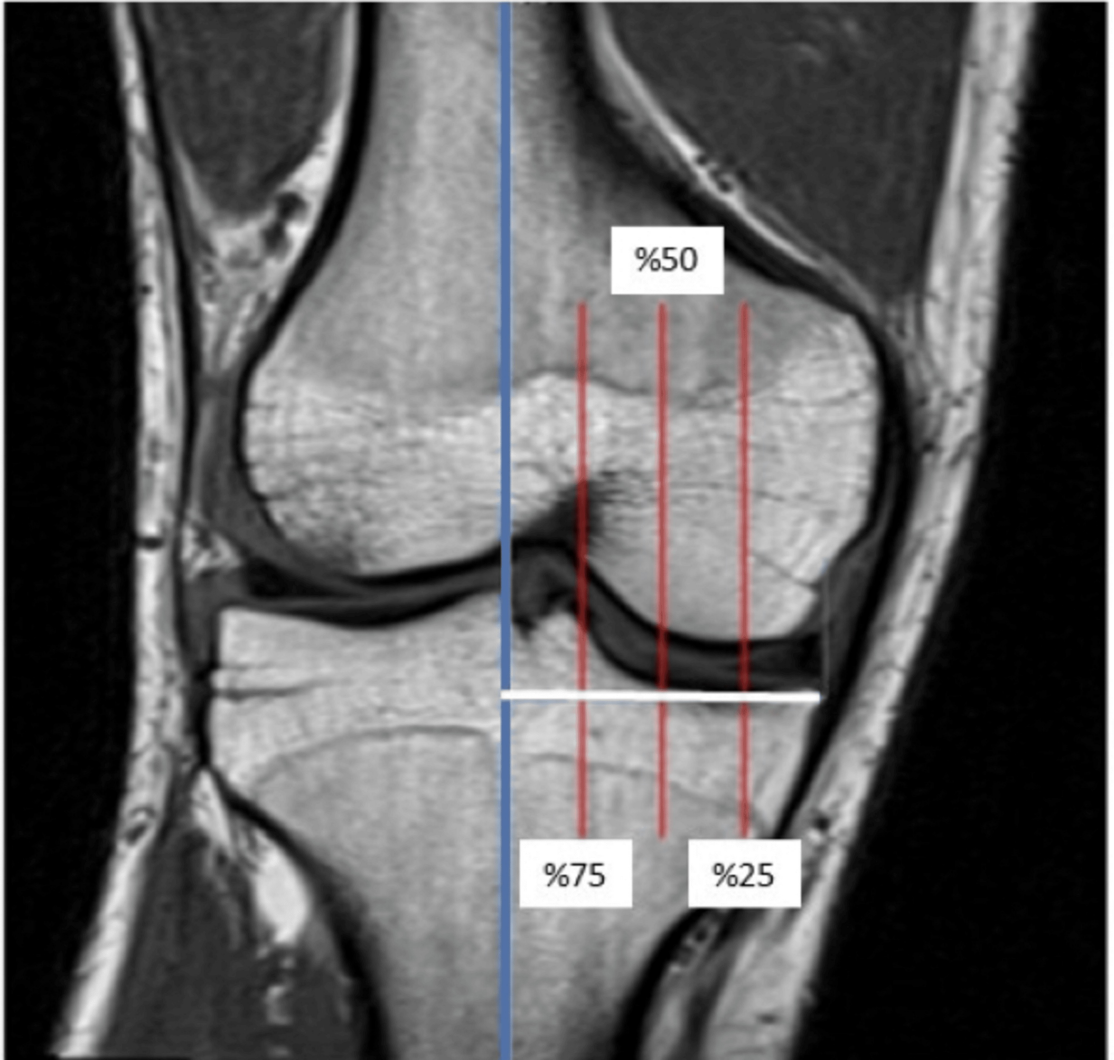
Determination of sections in MRI scans.

BMI, gender, age, and the side of the knee where the measurement was made were noted, and coronal, sagittal, and axial MRIs were transferred to Osirix Lite. MRI measurements for the posterior tibial and meniscus slope were conducted following a previously established method by Hohmann et al. [13]. First, the tibial plateau center was determined in the axial section of the transferred images. This fixed line was made visible in sagittal and coronal sections as well. The coronal section was used as the reference, with the vertical line in this section regarded as the center of the knee, and the tibial plateau of this line was divided into two parts, i.e., medial and lateral. Then, as a second line, a horizontal line was drawn from the lowest point of the tibial plateau from lateral to medial, the length of this line was recorded, and vertical parallel lines were drawn from the 25%-50% and 75% sections of this length (from outside to inside) (Figure 1).

**Figure 2:**
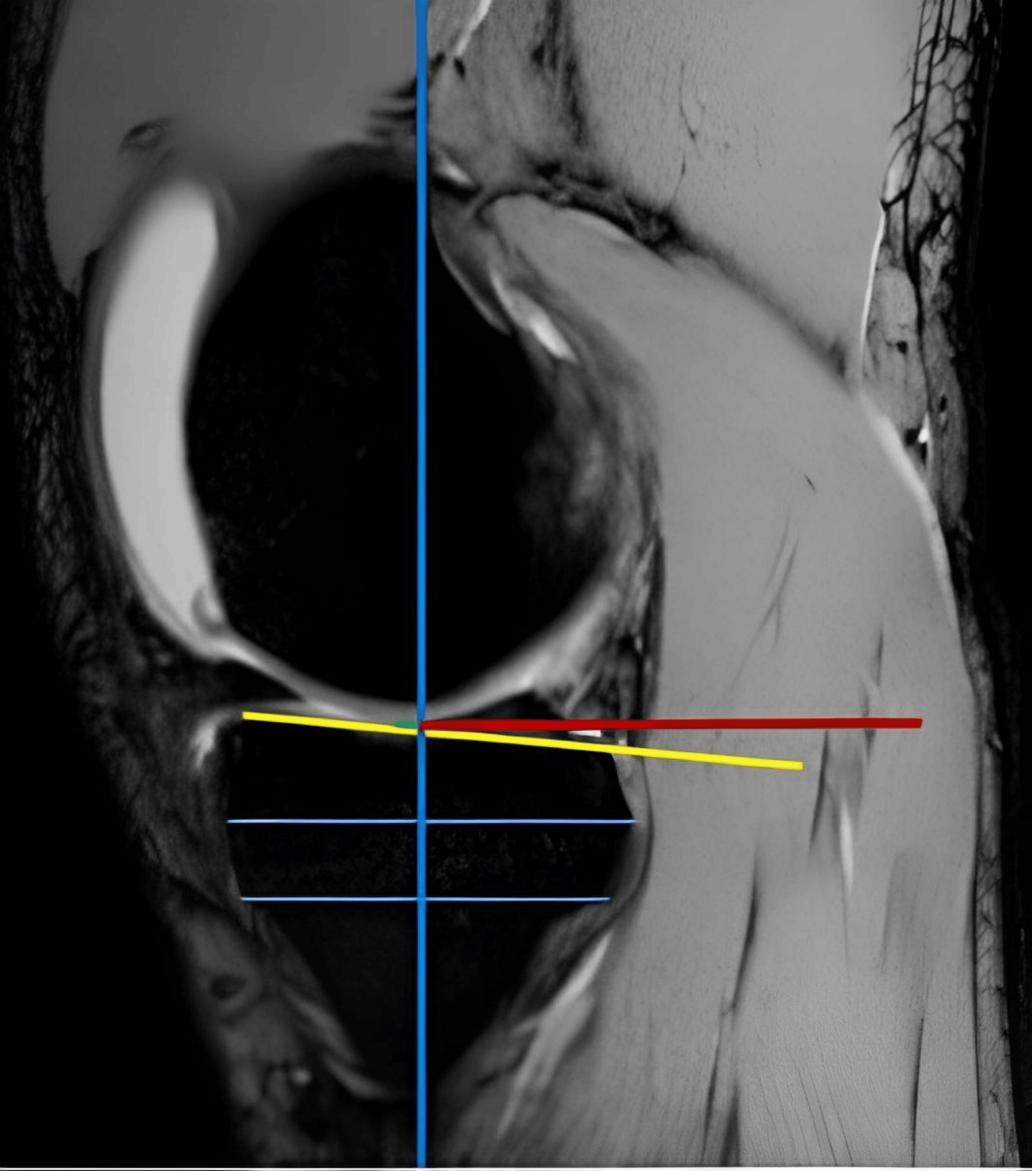
Tibial slope measurement in MRI scans.

In the next step, two horizontal lines were drawn on the sagittal section with a 2 cm interval from front to back. The vertical line connecting the midpoint of these two horizontal lines was marked as the posterior tibial anatomic axis (PTAA). The horizontal axis drawn from the most anterior to the most posterior on the sagittal section defined the tibial plateau. The angle between the horizontal axis drawn perpendicular to the PTAA and the tibial plateau was determined as the PTS angle (Figure 2).

A tangent line was drawn from anterior to posterior along the upper surface of the meniscus (medial and lateral), and the angle between this line and the tibial plateau line was recorded as the meniscus slope. The bone and meniscus slopes were mathematically added to calculate the combined slope. The slope data obtained from the medial and lateral knee compartments were compared with the patient’s BMI, age, and gender.

**Figure 3:**
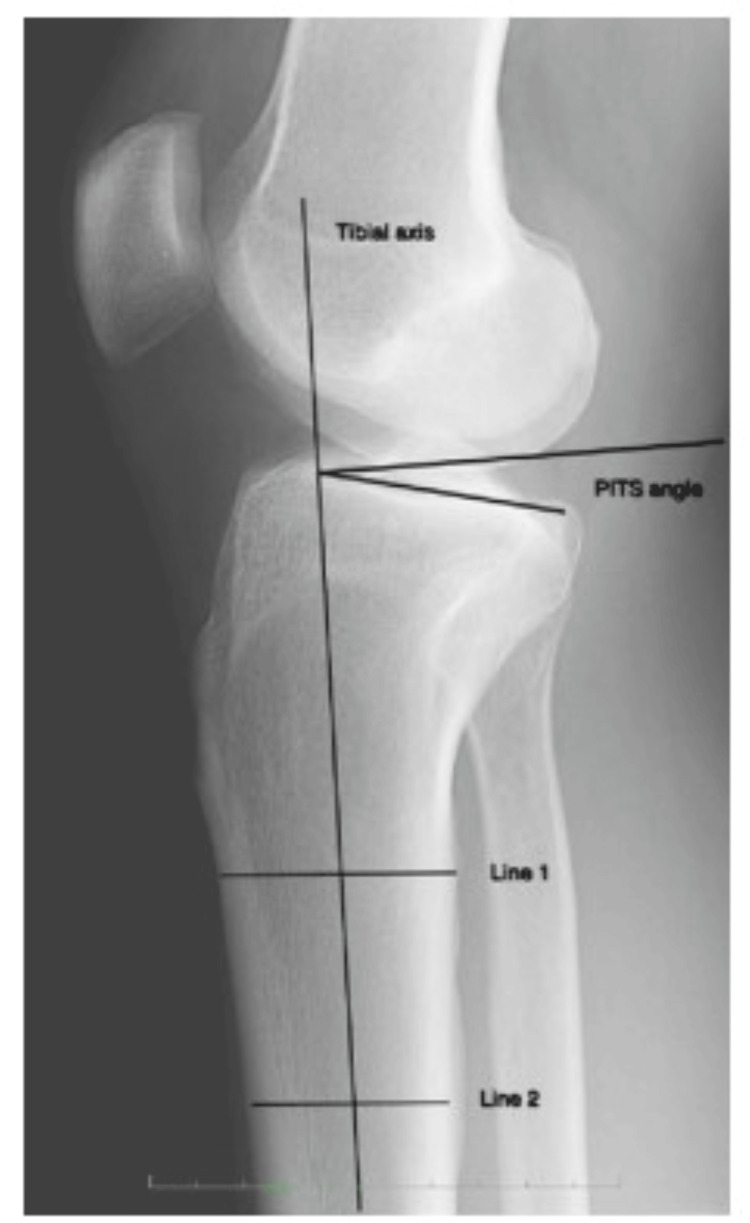
Tibial slope measurement on direct radiographs.

Direct knee radiographs of the patients were collected from the picture archiving and communication system. Two horizontal lines were drawn on the proximal tibia at 5 cm intervals on the radiographs, and the vertical line connecting the midpoints of these two horizontal lines was marked as the PTAA. The angle between a line tangent to the tibial plateau and the line perpendicular to the PTAA was recorded as the tibial slope (Figure 3).

Statistical analysis

Descriptive statistics for the study variables were calculated. Frequency and percentage for qualitative variables and arithmetic mean, standard deviation (SD), median, minimum, and maximum were calculated for quantitative variables. The Mann-Whitney U test was performed for statistical comparisons between two independent groups as the data deviated from parametric assumptions. The level of significance was accepted to be 0.05. SPSS (Demo Version 26.0 for Mac; IBM Corp., Armonk, NY, USA) was used for all statistical calculations.

## Results

Of the 66 MRI scans, eight revealed meniscal injuries, nine showed knee injuries, five had multiple ligament damage, and seven displayed osteochondral degenerative changes; five of these images were obtained from patients under 15 years old. As a result, 32 images were excluded, and 34 MRI scans from 32 individuals (13 females, 21 males) were included in the study. The ages of the individuals ranged from 15 to 78 (mean ± SD = 38.78 ± 16.87). In total, 17 (50%) of the images were of the right knee (female, 29.41%; male, 70.59%) and 17 (50%) of the left knee (female, 47.05%; male, 52.95%). Lateral radiographs and MRI scans of either the right or left knees of the individuals were examined. Participants were divided into the following two groups according to BMI: group I (BMI <24.99 kg/m^2^, n = 17), and group II (BMI >24.99 kg/m^2^, n = 17).

In the knee MRIs, the tibial slope, meniscus slope, and combined slope in the 25% sagittal sections of the medial compartment were measured as (mean ± SD) 1.75° ± 3.18°, 27.31° ± 1.41°, and 29.05° ± 3.80°, respectively. In the lateral compartment’s 25% sagittal sections, the tibial slope, meniscus slope, and combined slope were measured as (mean ± SD) 2.53° ± 2.82°, 28.08° ± 1.88°, and 30.62° ± 2.99°, respectively. The lateral meniscus slope and combined slope in the 25% section were statistically significantly higher compared to the medial meniscus slope in the same section.

In the 50% sagittal sections of the medial compartment, the tibial slope, meniscus slope, and combined slope were measured as (mean ± SD) 2.24° ± 2.93°, 27.7° ± 1.63°, and 29.94° ± 3.26°, respectively. In the lateral compartment, the tibial slope, meniscus slope, and combined slope were measured as (mean ± SD) 2.78° ± 2.56°, 28.72° ± 1.54°, and 30.87° ± 2.84°, respectively. No statistically significant differences were found between these results.

The tibial slope in the 75% sagittal sections of the medial compartment was measured as (mean ± SD) 1.36°± 3.22°, and in the lateral compartment as 2.62° ± 2.61°, with no statistically significant difference among them.

No statistically significant differences were found between the tibial, meniscus, and combined slope measurements in the medial and lateral compartments on MRIs in relation to BMI, age, and gender (Tables 1, 2). Direct radiograph measurements of the tibial slope did not show a statistically significant difference with age and gender (Table 3).

**Table 1:** The slopes of the medial and lateral knee compartments. Wilcoxon signed-rank test was used.

(%)		Medial compartment		Lateral compartment		
		Mean ± SD	Median	Mean ± SD	Median	P-value
Tibial slope	25	1.75 ± 3.18	3.02	2.53 ± 2.82	3.27	>0.05
	50	2.24 ± 2.93	3.05	2.78 ± 2.56	3.50	>0.05
	75	1.36 ± 3.22	2.80	2.62 ± 2.61	3.50	>0.05
Meniscus slope	25	27.31 ± 1.41	27.47	28.08 ± 1.88	28.25	0.05
	50	27.7 ± 1.63	28.00	28.72 ± 1.54	28.62	>0.05
Combined slope	25	29.05 ± 3.80	29.90	30.62 ± 2.99	31.12	0.011
	50	29.94 ± 3.26	30.10	30.87 ± 2.84	31.20	>0.05

**Table 2:** BMI: medial and lateral knee compartment slopes. BMI: body mass index; TS: tibial slope; MS: meniscus slope; CS: combined slope

Tibial and meniscus slope	BMI <24.99 kg/m^2^ (mean ± SD)		BMI >25 kg/m^2^ (mean ± SD)	
	Medial	Lateral	Medial	Lateral
%25 TS	1.26 ± 3.39	2.93 ± 2.34	2.23 ± 3.29	2.23 ± 2.98
%25 MS	27.36 ± 1.37	27.75 ± 1.83	27.25 ± 1.5	28.41 ± 1.94
%25 CS	28.63 ± 3.80	30.69 ± 2.46	29.49 ± 3.86	30.55 ± 3.52
%50 TS	1.81 ± 3.01	3.43 ± 1.87	2.47 ± 2.91	2.47 ± 2.91
%50 MS	27.51 ± 1.71	27.51 ± 1.71	28.07 ± 1.55	28.42 ± 1.94
%50 CS	29.33 ± 3.23	31.19 ± 2.51	30.55 ± 3.26	30.54 ± 3.18
%75 TS	0.66 ± 3.12	2.06 ± 3.25	2.93 ± 2.34	2.13 ± 3.25

**Table 3:** BMI and gender: tibial slopes on X-ray. BMI: body mass index; TS: tibial slope

	TS	
	Mean ± SD	Median (minimum-maximum)
BMI		
<24.99 kg/m^2^ (n = 17)	8.21 ± 1.18	24.02 (20.76-33.06)
>25 kg/m^2^ (n = 17)	7.71 ± 1.51	25.71 (22.49-31.2)
Total (n = 34)	7.96 ± 1.36	25.01 (20.76-33.06)
Gender		
Female (n = 13)	8.47 ± 1.49	8.8 (6.3-10.5)
Male (n = 21)	7.64 ± 1.2	7.8 (5.3-9.7)
Total (n = 34)	7.96 ± 1.36	7.85 (5.3-10.5)

## Discussion

The knee joint provides stability with static structures such as the joint capsule and ligaments and dynamic structures consisting of muscles and tendons. The kinematics and biomechanics of the knee joint are an interdisciplinary subject researched by scientific fields such as medicine, physical therapy, and rehabilitation [3]. The proximal tibia expands toward the lateral and medial plateaus. The geometric properties of the tibial plateau influence how loads are transmitted along the knee joint and are among the factors affecting joint biomechanics [4,14]. PTS is directly related to the anterior tibial translation of the articular cartilage, especially during weight-bearing of the trunk [14]. PTS plays a vital role in conditions such as ligamentum cruciatum anterior injury, total knee arthroplasty, and osteotomy [15].

Nunley et al. reported that the PTS was approximately 5°-6° [16], while other studies have established the normal PTS angle at 7° [17,18]. In our study, the PTS angle was found to be 7.64 ± 1.2 in males and 8.47 ± 1.49 in females, aligning with the values commonly accepted in the literature. In our study, no statistically significant difference was observed between the tibial slope measurements. However, Hohmann et al. reported a significant difference between the medial and lateral compartments at the 25% and 50% sections [13]. The difference in results may be attributed to the larger sample size in the study by Hohmann et al. In the retrospective study conducted by Cindemir et al. and Zhang et al., there was no relationship between PTS evaluated with MRI and gender [19,20]. In our study, consistent with previous research, the PTS angle measured by MRI showed no significant correlation with gender. Previous studies have found no statistically significant difference between age and PTS, and our findings were also consistent with these results [21,22]. In parallel with our results, the study conducted by Huang et al. reported that there was no significant difference between PTS and BMI [22]. A statistically significant difference between the medial meniscus slope and combined slopes at the 25% sagittal sections and the lateral meniscus slopes at the same sections was observed. In the study conducted by Hohmann et al., a statistically significant difference was reported between the medial combined slope and the lateral combined slope, but no significant difference was found between the medial meniscus slope and the lateral meniscus slope [13]. In the 50% section, no significant correlation was found between the medial meniscus slope and the lateral meniscus slope as well as between the combined slopes in the same sections. In the study done by Hohmann et al., no significant correlation was found between the medial and lateral meniscal slopes but a significant correlation between the combined slopes was reported [13]. In our study, no statistically significant difference was observed between tibial and meniscus slopes. Similarly, Hohmann et al. did not find a significant difference between these parameters in their research [13]. It has been reported that tibial and meniscus slopes are important factors for anterior cruciate ligament injuries and anterior-posterior stability of the knee joint [23,24]. In our study, no results were found to support that these factors have any effect on each other.

In this study, the measurement of the tibial slope using two different imaging techniques allowed for a clearer assessment of the tibial plateau. However, the small sample size limits the ability to make statistical inferences. Additionally, in the 75% sagittal sections, only tibial slope measurements were taken due to unclear images of the menisci.

## Conclusions

In knee pathologies, knee anatomy and kinesiology play a crucial role in planning treatment programs and interventions. The tibial bone provides fundamental functionality for weight transfer and stabilization in knee kinematics. It is widely accepted in the literature that various factors influence knee stabilization, one of which is the PTS. In our study, the factors that may affect the PTS were evaluated. The PTS angle should be evaluated by clinicians independently of age and gender for appropriate resection. Additionally, due to their roles in knee stability, we believe that the PTS and meniscal slope should be considered in the diagnosis and treatment process during rehabilitation.
